# *Salmonella enterica* Elicits and Is Restricted by Nitric Oxide and Reactive Oxygen Species on Tomato

**DOI:** 10.3389/fmicb.2020.00391

**Published:** 2020-03-13

**Authors:** Angela Marie C. Ferelli, Samantha Bolten, Brooke Szczesny, Shirley A. Micallef

**Affiliations:** ^1^Department of Plant Science and Landscape Architecture, University of Maryland, College Park, MD, United States; ^2^Centre for Food Safety and Security Systems, University of Maryland, College Park, MD, United States

**Keywords:** human pathogens on plants, nitrosative stress, oxidative stress, food safety, *Salmonella*–tomato interaction, nitric oxide, ROS

## Abstract

The enteric pathogen *Salmonella enterica* can interact with parts of the plant immune system despite not being a phytopathogen. Previous transcriptomic profiling of *S. enterica* associating with tomato suggested that *Salmonella* was responding to oxidative and nitrosative stress in the plant niche. We aimed to investigate whether *Salmonella* was eliciting generation of reactive oxygen species (ROS) and nitric oxide (NO), two components of the microbe-associated molecular pattern (MAMP)-triggered immunity (MTI) of plants. We also sought to determine whether this interaction had any measurable effects on *Salmonella* colonization of plants. Biochemical, gene expression and on-plant challenge assays of tomato vegetative and fruit organs were conducted to assess the elicitation of ROS and NO in response to *Salmonella* Newport association. The counter bacterial response and the effect of NO and ROS on *Salmonella* colonization was also investigated. We detected H_2_O_2_ in leaves and fruit following challenge with live *S.* Newport (*p* < 0.05). Conversely, NO was detected on leaves but not on fruit in response to *S*. Newport (*p* < 0.05). We found no evidence of plant defense attenuation by live *S.* Newport. Bacterial gene expression of *S.* Newport associating with leaves and fruit were indicative of adaptation to biotic stress in the plant niche. The nitrosative stress response genes *hmpA* and *yoaG* were significantly up-regulated in *S.* Newport on leaves and fruit tissue compared to tissue scavenged of NO or ROS (*p* < 0.05). Chemical modulation of these molecules in the plant had a restrictive effect on bacterial populations. Significantly higher *S.* Newport titers were retrieved from H_2_O_2_ scavenged leaves and fruit surfaces compared to controls (*p* < 0.05). Similarly, *S.* Newport counts recovered from NO-scavenged leaves, but not fruit, were higher compared to control (*p* < 0.05), and significantly lower on leaves pre-elicited to produce endogenous NO. We present evidence of *Salmonella* elicitation of ROS and NO in tomato, which appear to have a restricting effect on the pathogen. Moreover, bacterial recognition of ROS and NO stress was detected. This work shows that tomato has mechanisms to restrict *Salmonella* populations and ROS and NO detoxification may play an important role in *Salmonella* adaptation to the plant niche.

## Introduction

Non-typhoidal *Salmonella enterica* is a leading cause of foodborne illness transmitted by fresh fruit and vegetables (Callejón et al., 2015). Traceback investigations of several salmonellosis outbreaks implicating fresh produce have pointed to contamination sources in crop production areas (Greene et al., 2008; Bennett et al., 2015). *S. enterica* is frequently isolated from water and soil in agricultural settings (Micallef et al., 2012; Bell et al., 2015; Callahan et al., 2019), suggesting that this enteric pathogen is able to cycle through various ecological niches and become established in the plant phyllosphere.

*Salmonella enterica* can survive and multiply on plants (Brandl and Mandrell, 2002), the success of which is influenced by multiple factors (Brandl et al., 2013), including plant genotype and organ (Barak et al., 2011; Han and Micallef, 2014), age (Brandl and Amundson, 2008; Zheng et al., 2013), surface metabolite profiles (Han and Micallef, 2016), as well as resident epiphytes (Poza-Carrion et al., 2013). Studies on *S. enterica* colonizing tomato fruit wounds, lettuce soft rot lesions and sprouts have identified specific sets of genes expressed under these conditions, including genes involved in amino acid biosynthesis, fatty acid metabolism, iron acquisition, attachment and stress response (Goudeau et al., 2013; Salazar et al., 2013; Tan et al., 2016; de Moraes et al., 2017, 2018). Research within our group investigating gene expression in *S. enterica* epiphytically colonizing tomato shoots and roots detected several genes involved in nitrosative and oxidative stress mitigation and multiple *Salmonella* pathogenicity island-2-encoded type III secretion system genes (T3SS-2) (Han et al., unpublished). These findings pointed to interplay between the enteric pathogen and the plant, as a result of tomato plant recognition and immune response.

Plants recognize potential microbial pathogens via MAMP interaction with pathogen recognition receptors (PRRs) (Jones and Dangl, 2006). This recognition initiates several strong yet transient signaling events to occur, beginning with an influx of calcium ions into the cell (Ranf et al., 2011) which induces a burst of reactive oxygen species (ROS) (Doke, 1983) and nitric oxide (NO) (Ma et al., 2008; Rasul et al., 2012). The generation of ROS and the more recently identified NO serve multiple purposes for the plant. The ROS burst can directly control the potential pathogen threat and, together with NO, may activate mitogen associated protein kinases (MAPKs) and signal the up-regulation of transcription factors that initiate transient defense responses. These include salicylic acid (Tsuda et al., 2008) and ethylene biosynthesis (Liu and Zhang, 2004) which in part comprise MAMP- triggered immunity (MTI) (Meng and Zhang, 2013). The non-plant pathogen *S.* Typhimurium and its flagellin 22 (flg22) have been shown to induce an ROS burst in tobacco and tomato leaf disks, respectively (Shirron and Yaron, 2011; Meng et al., 2013). Flagellin 22 from *S*. Typhimurium was recognized by tobacco and *Arabidopsis thaliana* through the FLS2 receptor, inducing MTI which was effective in restricting *S. enterica* and the plant pathogen *Pseudomonas syringae* pv. *tomato* (Meng et al., 2013; Garcia et al., 2014). The role of NO in plant defense is less understood but it was generated in *Arabidopsis* in response to lipopolysaccharide (LPS) challenge (Zeidler et al., 2004) and also required for abscisic acid-induced stomatal closure (Neill et al., 2002). Melotto et al. (2006) showed that *Arabidopsis* guard cells generated NO in response to flg22 and LPS, which was followed by stomatal closure. They also reported that *S. enterica* was able to trigger stomatal closure in *Arabidopsis*. Furthermore, flagellar mutants of *S*. Typhimurium were shown to better colonize wheat, alfalfa and *Arabidopsis*, suggesting that attenuation of MAMPs favored bacterial colonization (Iniguez et al., 2005). Finally, some studies have suggested that *S. enterica* may have the ability to suppress MTI in *Arabidopsis* and tobacco and the role of effector proteins has been invoked (Shirron and Yaron, 2011; Garcia et al., 2014; Neumann et al., 2014).

The current state of knowledge and our finding that *S. enterica* expresses oxidative and nitrosative stress genes, and T3SS-2 genes when colonizing tomato shoot and root surfaces (Han et al., unpublished), implied that *S. enterica* recognition by tomato induces the generation of both ROS and NO, and that *S. enterica* attempts to attenuate the plant immune response. We set out to test this hypothesis in leaves, but also more relevantly, on tomato fruit, in relation to both live and killed *S. enterica* to assess potential plant defense suppression. MTI induction in tomato leaves and fruit in response to *S. enterica* Newport (*Se*N) association was investigated by measuring NO and ROS generation. Moreover, we sought to describe the effect of this plant response on *S. enterica* populations. The reciprocal bacterial response and the effect of surface modulation of NO and ROS on *S. enterica* colonization of tomato leaves and fruit was also evaluated.

## Materials and Methods

### Cultivation of Plant Material

Tomato seeds cv. ‘Heinz-1706’ were obtained from the Tomato Genetics Resource Center (TGRC) from the University of California, Davis. After pre-treatment in 30% w/v polyethylene glycol solution at room temperature with shaking for 72 h, seeds were germinated in potting media (Sunshine LC1; Sungro Horticulture, Canada) at 25°C. Germinated seeds were transferred to fresh potting media supplemented with fertilizer (Osmocote controlled release fertilizer 18–6–12:nitrogen–phosphate–potash, The Scotts Company LLC., Marysville, OH, United States) and subjected to a 16 h-light/8 h-dark photoperiod and 26°C day temperature/18°C night temperature with 70% humidity (RH) at the University of Maryland Research Greenhouse. Plants were drip irrigated. Tomato seedlings were grown to five true leaves before experimentation, unless otherwise noted. Seedlings for experimentation were transported to a BSL-2 growth chamber (16 h-light/8 h-dark photoperiod and 23°C constant temperature with 70–80% RH) at least 5 days prior to inoculation and flood irrigated to a depth of 5 cm in trays every 4 days. Water was withheld 3 h before inoculating leaves and for the duration of experiments. For fruit, plants were either grown in the field (summer) at the Wye Research and Education Centre, Queenstown, MD, United States, or transplanted into 6 L pots to be grown in the greenhouse (winter) once they reached the 5-leaf stage. In the greenhouse, plants were fertilized once a week and treated with non-organophosphate containing pesticide once every 2 weeks for aphid and white fly management. Once plants reached maturity, red, ripe fruit was collected immediately prior to experimentation, rinsed with sterile water and air dried, unless otherwise stated.

### Bacterial Strains

The *Salmonella enterica* Newport strain used was an environmental isolate collected from an irrigation pond that matched a recurring tomato outbreak strain (Greene et al., 2008). *Se*N had been previously adapted to rifampicin (rif) and was therefore maintained at −80°C in Brucella Broth (BD, Sparks, MD, United States) containing 15% glycerol and 50 μg/mL rifampicin (rif, Tokyo Chemical Industry, Portland, OR, United States). For each experiment, cultures of *Se*N were grown overnight on Trypticase Soy Agar (TSA; BD) + rif at 35°C. A single colony was selected, suspended in sterile water, and diluted to OD_600_ = 0.34 – approximately 8.5 log CFU/mL. Serial dilutions were made in sterile water for inoculum preparation and in 0.1% peptone water for bacterial quantification (BD Difco, Sparks, MD, United States). Cells were enumerated on TSArif. *Pseudomonas syringae* pv. *maculicola* ES4326 (*Psm*), a relative to *P. syringae* pv. *tomato* with similar virulence and in the same genospecies clade (genospecies III) (Preston, 2000) was grown in TSA at 25°C, and inoculum prepared as described above.

### Detection of ROS in Leaves and Fruit

To detect the amount of H_2_O_2_ produced in leaves following *Se*N challenge, 3,3′-diaminobenzidine (DAB) staining was adapted from Bindschedler et al. (2006). Briefly, a third emerged leaflet from freshly watered, 5-leaved ‘Heinz-1706’ plants was syringe infiltrated into the abaxial surface with 500 uL of either *Se*N in sterile water at 8 log CFU/mL, heat-killed *Se*N, or sterile water (control) (*N* = 6 plants per treatment). Positive controls were conducted with *Psm*. Inoculated plants were incubated in a BSL-2 growth chamber. All experiments were performed in a complete randomized design (CRD) and repeated at least twice. At 0.1 and 24 hours post-inoculation (hpi), inoculated leaflets were excised and submerged in 5 mL DAB solution (1 mg/mL aqueous DAB (Alfa Aesar, Ward Hill, MA, United States), 200 mM Na_2_HPO_4_ (VWR, West Chester, PA, United States), 0.05% Tween 20 (Amresco, Solon, OH, United States) and 100 μL 3 N HCl). Samples were vacuum-infiltrated in a vacuum desiccator attached to the laboratory vacuum system for 4 min, then incubated in the dark at 23°C with shaking at 50 rpm for 4 h. At the end of staining, decolorizer solution was added (3:1:1 95% ethanol, glycerol, glacial acetic acid) and samples were incubated in a boiling water bath for 15 min. Decolorized leaflets were fixed to paper and imaged with an Epson V330 photo scanner. The stain, corresponding to H_2_O_2_ production, was analyzed for intensity via ImageJ2 FIJI package (Schindelin et al., 2012). Optical density in leaves was calculated using the formula

$$O\,D=\log_{10}\left(\max i\,n\,t\,e\,n\,s\,i\,t\,y÷m\,e\,a\,n\,i\,n\,t\,e\,n\,s\,i\,t\,y\,o\,f\,l\,e\,a\,f\,a\,r\,e\,a\right).$$

To detect a range of ROS produced from *Se*N challenge on fruit, staining with 6-chloromethyl-2′,7′-dichlorodihydrofluorescein diacetate, acetyl ester (CM-H_2_DCFDA; Invitrogen, Molecular Probes Inc., Eugene, OR, United States) was adapted from Shin and Schachtman (2004). Briefly, 3 mm × 3 mm sections of ripe tomato exocarp per fruit were excised with a sterile razor and placed in separate black 96-well plates to serve as technical replicates for one fruit (Corning, Nazareth, PA, United States). Aliquots of 150 μL deionized water were delivered to the sample wells and incubated overnight in the dark at 4°C to allow for dissipation of any ROS production due to injury. Immediately before experimentation, wells were washed with 100 μL sterile water. One hundred micro-liters of 8.0 log CFU/mL *Se*N, heat killed *Se*N (14 h only) or sterile water were delivered to sample wells (*N* = 4 fruit per treatment, with three technical replicates per fruit). Samples were vacuum-infiltrated in a vacuum desiccator for 5 min, then shaken at 100 rpm at 27°C for 3 or 14 hpi. At the time of sampling, 25 μM CM-H_2_DCFDA in water was added to each well. The fluorophore was allowed to react for 30 min at 23°C in the dark with shaking at 50 rpm before being imaged with a Synergy HTX Microplate reader (BioTek, Winooski, VT, United States) at 485 nm excitation, 520 nm emission with 50 gain.

### Detection of NO in Leaves and Fruit

To measure amounts of NO release from tomato when challenged with *Se*N, 4,5-diaminofluorescein diacetate (DAF-2 DA; Fisher Scientific, Hampton, NH, United States) was used for its ability to complex intercellular NO as well as NO in solution (Rasul et al., 2012). For measurements on leaves, leaflets of mature ‘Heinz-1706’ plants grown in the research greenhouse were punched three times with a 3 mm hole puncher and cut tissue pieces were placed in separate wells in a black 96-well plate, to serve as technical replicates for each leaf (Corning, Nazareth, PA, United States) (*N* = 5 leaves per treatment, with three technical replicates per leaf). For measurements on fruit, 3 mm × 3 mm sections of ripe tomato exocarp were excised with a sterile razor (*N* = 5 fruit per treatment, with three technical replicates each). For both experiments, 150 μL deionized water was placed in the sample wells and plates were incubated overnight in the dark at 4°C to allow for the dissipation of injury related NO signal. All experiments were performed in a CRD and repeated at least twice. Prior to inoculation, tissues were washed twice with sterile water, then challenged with 100 μL of 8 log CFU/mL *Se*N, heat killed *Se*N, sterile water or 8 log CFU/mL *Psm.* Samples were vacuum-infiltrated for 5 min, then shaken at 100 rpm at 27°C. At 0.1, 1, and 3 h a final concentration of 15 μM DAF-2 DA in 50 mM Tris HCl pH 7.5 was delivered to the wells. Plates were incubated in the dark for 30 min at 27°C with shaking at 50 rpm and immediately read on the Synergy HTX (BioTek) at 485 nm excitation, 520 nm emission with 50 gain.

### Targeted q-RT-PCR of *Se*N Genes Colonizing Leaf and Fruit Surfaces

To evaluate genes involved in nitrosative and oxidative stress responses in *Se*N colonizing the tomato phyllosphere, 3-leaf tomato seedlings cv. ‘Heinz-1706’ were pre-treated with water (native environment), 2-4-carboxyphenyl-4,4,5,5-tetramethylimidazoline-1-oxyl-3-oxide (cPTIO; NO-limiting environment) or CaCl_2_ (excess NO environment), then challenged with *Se*N. To achieve this, 48 plants grown in autoclaved LC-1 potting media (Sunshine LC1) were separated into three groups and aerosol-sprayed with either 1 mL 0.5% CaCl_2_, ddH_2_O or 0.2 mM cPTIO. The plants were allowed to air-dry for 30 min. The second emerged leaf was challenged with 7 log CFU/mL *Se*N, delivered as ten 2-μL spots onto the leaf surface. Plants were incubated in the BSL-2 growth chamber, as previously described. At 6 hpi, inoculated leaves of three plants were pooled to comprise one composite sample (*N* = 4 composite samples per treatment). Samples were immediately fixed in 2:1 RNAProtect Bacteria Reagent (Qiagen, Germantown, MD, United States):ddH_2_O. Samples were sonicated on a 8510 Branson Sonicator at full strength for 2 min to dislodge surface attached bacteria. The wash solution containing *Se*N was transferred to a fresh tube and processed as described below.

To evaluate the role of genes involved in colonization of tomato fruit, cv. ‘Heinz-1706’ mature red fruit were washed with 200 ppm sodium hypochlorite and triple rinsed with ddH_2_O. Fruit were then syringe-injected at the calyx with either 500 μL ddH_2_O or 0.25 mM ascorbic acid. Seven log CFU/mL *Se*N was delivered as five 20-μL spots on the fruit surface. Fruit were incubated in the BSL-2 growth chamber in identical conditions as seedlings. At 6 hpi, five fruit from each treatment were pooled to comprise one composite sample (*N* = 4 composite samples per treatment). Samples were placed in RNA*later* Stabilization Solution (Invitrogen, Carlsbad, CA, United States). Fruit were vigorously vortexed for 3 min to dislodge attached cells. The wash solution containing *Se*N was transferred to a fresh tube and processed as described below.

In both experiments, 0.5 mL *Se*N inoculum in water was immediately fixed with RNAProtect Bacteria Reagent or RNA*later* Stabilization Solution to serve as the baseline for gene expression. All samples were centrifuged at 5,000 × *g* for 30 min. Total RNA was extracted from the resultant pellet using the Qiagen RNeasy Mini kit (leaves) (Qiagen) or the Purelink RNA Isolation kit (fruit) (Invitrogen) with 45 min on-column DNA digestion (Invitrogen). Resulting total RNA was evaluated on the Nanodrop 1000 (Thermo Fisher) for quality. PCR of target genes was performed using 1 μL total RNA sample template to ensure depletion of gDNA. cDNA was synthesized with Verso cDNA kit (Thermo Scientific, Waltham, MA, United States) and 1 ng samples were subjected to q-PCR of genes using primers listed in Table 1. Primers were used at 100 nM concentration and verified to be 90–105% efficient. Plant material was verified to produce no off-target amplification before experimentation. In a series of experiments using TSB amended with treatment reagents, *Se*N gene expression was confirmed to be reflective of epiphytic habit on tomato surface and not an artifact of interaction with elicitor or scavenger (Supplementary Figure 1). Amplification was conducted on an ABI Step-One Plus (Applied Biosystems, Foster City, CA, United States) with SYBR as a reporter (PowerUP^TM^ SYBR Green Master Mix, Thermo Fisher Scientific, Austin, TX, United States) using the following parameters: 50°C for 2 min, 95°C for 2 min, followed by 40 cycles of 95°C for 15 s and 59°C for 30 s. Melt curve analysis was included to ensure product specificity. The cutoff Ct was set to 36.5 cycles. Data were analyzed on the ABI Step One Plus instrument with the ΔΔCt method (Pfaffl, 2001) using RNA polymerase sigma factor *rpoD* as the endogenous control. Relative gene expression was compared to expression in *Se*N inoculum after internal normalization to *rpoD* expression.

**TABLE 1:** List of genes, their qPCR primers and efficiencies used to examine *S.* Newport gene expression on tomato leaves and fruits.

Functional category	Gene	Function	5′–3′ sequence	qPCR primer source	Efficiency
ROS response	*ahpC*	Peroxiredoxin	TCGCTTCGCCTTCTTTCCAT GACCTTTGTTGTTGACCCGC	This study	101%
	*katG*	Catalase	GACTCACCGACACCCTGAAG CACGGTCTCTTCGTCGTTCA	This study	102%
NO response and detoxification	*hmpA*	Flavohemoglobin, nitric oxide dioxygenase activity	GAACATTTCGTCCAGCGTCG ATCAGCGTGAAGCCCTGTTT	This study	95%
	*yoaG*	Cytoplasmic protein in NsrR regulon	ATAGCAACGGCGTCTCTGTG GGTATCGTAGGAACGCACGG	This study	101%
Virulence	*phoP*	Virulence regulator	CGACTTTATCCTGCCAGCCT GCCTTTCCTTAATACGCCGC	This study	91%
	*phoQ*	Virulence regulator, membrane-bound sensor kinase	TATGGTGTGGAGCTGGTTCG CGGCGATCCACAGTAAAGGA	This study	91%
	*sdiA*	Virulence regulator, quorum-sensing regulator	GATGAGGTCTTCCCTTCCGC TACGCTGCTCCTCGTTTACC	This study	90%
Environmental fitness	*marA*	DNA-binding transcriptional activator for antibiotics resistance operon MarRAB	TACGGCTGCGGATGTATTGG CGAGGATAACCTGGAGTCGC	This study	105%
	*nmpC*	Outer membrane porin protein, cell wall biogenesis	GTCCGTCCATCGCTTACCTG GCTTTGGTGAAGTCGCTGTC	This study	94%
	*trpE*	Tryptophan biosynthesis protein	CGCTTTTTCACCAGGTCTGC	This study	102%
			AACGCCTGAATGGTGACAGT		
Housekeeping	*rpoD*	RNA polymerase sigma factor	GTGAAATGGGCACTGTTGAACTG	Karlinsey et al. (2012)	101%
			TTCCAGCAGATAGGTAATGGCTTC		

### Modulation of Endogenous Hydrogen Peroxide and Nitric Oxide Levels, and Plant Colonization Assays

To investigate the effect of plant derived ROS and NO on *Se*N survival on tomato surfaces, the third emerged leaf on 5-leaved ‘Heinz-1706’ seedlings or mature fruit were treated with reagents to either scavenge surface ROS (Bradley et al., 1992; Lee et al., 1999) or NO (Małolepsza and Różalska, 2005; Keshavarz-Tohid et al., 2016), or elicit production of NO (Chakraborty et al., 2016), then subsequently inoculated with *Se*N. The reagents employed, concentrations and application methods for leaves and fruit are detailed in Table 2. All experiments were performed in a CRD and repeated at least twice. After application of cPTIO and CaCl_2_, fruit (*N* = 10 for each treatment) and leaves (*N* = 18 for cPTIO experiments, *N* = 4 for CaCl_2_ experiments) were left to air-dry for 4 h at room temperature. Ascorbic acid-treated leaves (*N* = 3 per treatment) were left to dry for 2 h and fruit (*N* = 11 per treatment) were left to dry for 30 min prior to *Se*N inoculation. Following pretreatment, a suspension of 5.5 log CFU/mL *Se*N in water was applied to the surface of leaflets or fruit in ten 2-μL spots. Samples were incubated at 23°C at 75% RH for 12 h. To retrieve viable *Se*N, inoculated leaflets or fruit exocarp were cut and excised, respectively, as previously described, and diluted in 0.1% peptone water. Leaflets were hand-massaged and sonicated and fruit were hand-massaged and vortexed for 2 min before serially plating dilutions onto TSArif and incubating at 35°C for 20 h.

**TABLE 2:** Chemicals and application methodology for modulation of tomato leaf and fruit NO, ROS levels.

Pretreatment purpose	Tissue	Chemical	Source	Application method
H_2_O_2_ scavenger	Fruit	0.25 mM ascorbic acid	Sigma, St. Louis, MO, United States	Calyx syringe injection
	Leaves	2.5 mM ascorbic acid	Sigma, St. Louis, MO, United States	Abaxial syringe infiltration
NO scavenger	Leaves and fruit	0.22 mM 2-4-carboxyphenyl-4,4,5,5-tetramethylimidazoline-1-oxyl-3-oxide (cPTIO)	Enzo Life Sciences, Farmingdale, NY, United States	Adaxial aerosol spray
NO elicitor	Leaves and fruit	0.5% Calcium Chloride (CaCl_2_)	Sigma, St. Louis, MO, United States	Adaxial aerosol spray

### Statistical Analysis

Statistical analysis was conducted in JMP version 14.1.0. The degree of NO and ROS elicitation was analyzed for significance via Student’s *t-*test in pairwise comparisons, or ANOVA and orthogonal contrasts for *a priori* comparisons excluding the positive control (α = 0.05). Targeted gene expression data was analyzed for significance using ANOVA and *post hoc* via Dunnett’s test (inoculum control versus on-plant gene expression) or Tukey’s Honestly Significant Difference (cPTIO versus CaCl_2_ versus control on leaves), and Student’s *t*-test (ascorbic acid versus control on fruit) all at α = 0.05. For on-plant challenge assays, Student’s *t*-test was employed to compare treatment to water control (α = 0.05).

## Results

### *Salmonella* Newport Elicits H_2_O_2_ Production in Tomato Leaves and Fruit

**FIGURE 1:**
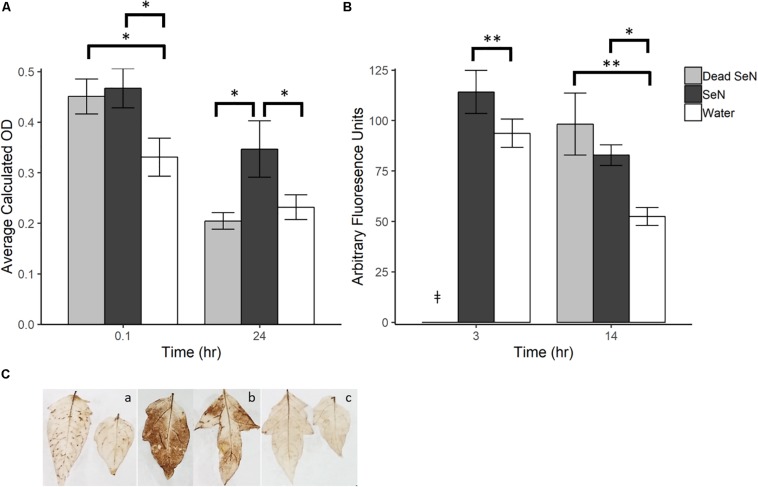
Plant derived ROS produced following challenge with sterile water (H_2_O), heat killed *S*. Newport (dead *Se*N) or live *S.* Newport. **(A)** Optical density showing degree of DAB stain in tomato leaves following *Se*N challenge via syringe infiltration. **(B)** CM-H_2_DCFDA fluorescence from tomato fruit peel challenged with *Se*N. **(C)** DAB staining of leaflets 24 h post-inoculation with (a) *Pseudomonas syringae* (*Psm*), (b) *Se*N, and (c) water. Error bars represent standard error of the mean and ^∗∗^ *p* < 0.01; ^∗^*p* ≤ 0.05. ^‡^denotes treatment was not included in that timepoint.

A dark brown precipitate indicative of H_2_O_2_ production was detected immediately following inoculation of leaves with *Se*N (0.1 hpi) (Figures 1A,C). At 0.1 hpi, the brown precipitate deposited in *Se*N-challenged leaves (0.47 ± 0.10; *p* < 0.05) and heat-killed *Se*N-challenged leaves (0.45 ± 0.08; *p* < 0.05) was significantly darker than the negative control. Twenty-four hpi, heat-killed *Se*N (0.20 ± 0.05) and water control (0.23 ± 0.08) had comparable measurements, while leaves treated with live *Se*N exhibited darker staining (0.35 ± 0.18; *p* < 0.05) compared to control. The staining obtained with *Se*N was diffuse, as opposed to localized in spots as was observed for the virulent *Psm* pathogen (Figure 1C). Overall, we found no evidence of live *Se*N suppression of ROS generation.

In fruit, at 3 h post-challenge, more ROS was detected in live *Se*N-treated exocarp than in water-treated exocarp (*p* < 0.01; Figure 1B). At 14 hpi, significantly more ROS was detected in exocarp samples treated with heat-killed (98.18 ± 51.0 Arbitrary fluoresence units, Au; *p* < 0.01) and live *Se*N (82.82 ± 17.0 Au; *p* < 0.05) compared to controls (51.41 ± 15.5 Au). Conversely to leaves, heat-killed S*e*N produced a similar signal to live S*e*N in fruit at the later timepoint. Taken together, these measurements suggest that live *Se*N can induce ROS generation in both tomato leaf and fruit exocarp tissue. Further, the differential ROS levels detected in tomato leaves inoculated with live or heat-killed *Se*N showed that ROS generation was more prolonged in response to live cells, potentially triggered by *Salmonella* activity in the leaf niche, and not solely from interaction with microbial surface cellular components.

### NO Production Was Detected in Leaves but Not in Fruit Exocarp Challenged With *Se*N

**FIGURE 2:**
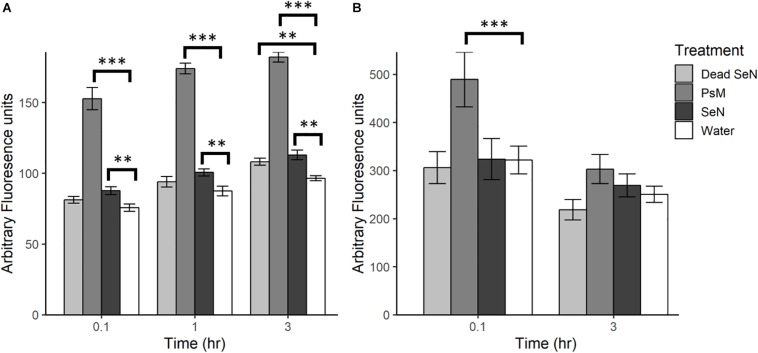
Plant derived NO produced following challenge with heat killed *S*. Newport (dead *Se*N), *Pseudomonas syringae* (*Psm*) or *S.* Newport (*Se*N) on **(A)** tomato leaves and **(B)** tomato fruit, measured with DAF-2 DA. Error bars represent standard error of the mean and ^∗∗^*p* < 0.01; ^∗∗∗^*p* ≤ 0.001.

Following leaf surface inoculation with *Se*N, NO was detected in all treatments (Figure 2). The tomato pathogen *Psm*-treated leaf sections served as a positive control and, as expected, induced significantly more NO than the water control at all sampling times (*p* < 0.001). At each timepoint, live *Se*N induced a stronger signal in leaves compared to water (87.67 ± 13.24, 100.6 ± 13.4 and 112.9 ± 18.8 Au, respectively; all *p* < 0.01; Figure 2A). Heat-killed *Se*N also induced NO at 3 hpi (*p* < 0.01). We did not observe any evidence of live *Se*N suppression of NO generation.

Fruit tissue fluorescence for all treatments decreased over time by an average of 100.1 Au, with the smallest average decrease observed in live *Se*N-treated fruit tissue and the largest change in *Psm* treated leaf sections (Figure 2B). *Psm* produced significantly more NO than the water control at 0.1 hpi (*p* < 0.001) but not at 3 hpi. No significant exocarp production of NO was detected in *Se*N-treated fruit exocarp compared to the water control, either at 0.1 or 3 hpi.

Overall, fruit exocarp tissue produced a stronger NO signal than leaf tissue (*p* < 0.05) regardless of treatment. If NO was produced in relation to SeN, it was not strong enough to be detected with the method employed over the background NO being generated. Further, exocarp measurements exhibited a larger variation with a coefficient of variation (CV) of 0.52 compared to 0.36 for leaf sections. Taken together, these data suggest that live *Se*N can induce NO generation in tomato leaves, but not fruit.

### Expression of ROS and NO Detoxification Genes Was Detected in *Se*N Colonizing Leaf Surfaces

**FIGURE 3:**
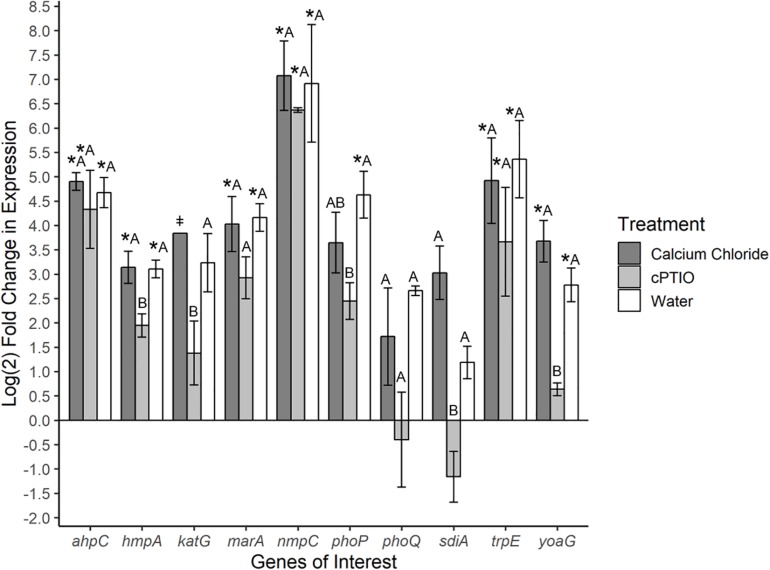
Log_2_-fold change in expression of *S.* Newport genes compared to expression in inoculum. ΔΔCt results of *S.* Newport colonizing 3-leaf tomato seedlings pretreated to reflect the native environment (H_2_O), NO-limiting (cPTIO) or NO-excess (CaCl_2_) environments normalized against expression of the sigma factor *rpoD. N* = 4 groups of three pooled plants per treatment. Error bars represent the standard error of the mean (SEM). ^‡^ denotes insufficient data to generate (SEM). Asterisks denote significance in gene transcription compared to inoculum according to Dunnett’s test (α = 0.05). Letters denote significant differences in gene transcription among treatments for each target gene using Tukey’s Honestly Significant Difference (α = 0.05).

To investigate specific bacterial responses to the observed elicited H_2_O_2_ and NO on tomato leaves, a targeted gene expression analysis was conducted on *Se*N cells inoculated onto native leaves, or leaves pre-treated to enhance or limit NO. We assayed genes responsible for NO detoxification, ROS mitigation and other environmental fitness factors (Table 1) that were previously found to be expressed in *S. enterica* Typhimurium colonizing tomato shoots and roots (Han et al., unpublished). On leaves, 78% of *Se*N samples displayed ≥|2| -fold change in gene expression compared to the inoculum. The flavohemoglobin *hmpA*, a main detoxifier of NO in oxygenated environments (Crawford and Goldberg, 1998) and *yoaG*, a cytoplasmic protein in the NsrR regulon (Lin et al., 2007), were shown to be differentially up-regulated in NO-excess and native leaves compared to the inoculum and to NO-limiting treatments (*p* < 0.05) (Figure 3). Compared to the inoculum, expression of *hmpA* increased threefold (*p* < 0.05) in *Se*N associating with native leaves and NO-excess leaves. Additionally, the *yoaG* gene showed an almost threefold increase on native leaves and a fourfold increase on NO-excess leaves (*p* < 0.05) compared to inoculum expression levels. The similar increases in gene expression in both NO-excess and native leaves, relative to the inoculum, suggest that *Se*N itself is serving as a strong NO elicitor on leaves. Finally, gene expression of both *hmpA* and *yoaG* in NO-limiting environments was comparable to expression levels in the inoculum. Taken together, these findings suggest that *Se*N may be countering NO and/or NO-regulated plant responses.

The gene *ahpC* encodes an enzyme alkyl hydroperoxide reductase (Ahp) that protects cells from oxidative stress by catalyzing the reduction of hydrogen peroxide and organic peroxides (Seaver and Imlay, 2001). This gene was up-regulated in all treatments compared to inoculum (*p* < 0.05), but was not found to be differentially expressed among treatments. Expression of the catalase gene *katG* (Morgan et al., 1986) was not different from the inoculum, but differed between *Se*N on native versus cPTIO-treated leaves (*p* = 0.053). Overall, *Se*N appeared to be responding to oxidative stress on leaves.

The virulence factors *phoQ* two component system (Monsieurs et al., 2005) and the quorum sensing gene *sdiA* (Ahmer et al., 1998) both exhibited an increase in expression on native and NO-excess environments. The transcription of *sdiA* on CaCl_2_-treated leaves and of *phoQ* on cPTIO-treated leaves was higher and lower, respectively, than transcription in the inoculum but weakly statistically supported (*p* = 0.06). Compared to expression on NO-limiting environments, *sdiA* expression increased fourfold on NO-excess leaves and *phoQ* expression increasing threefold on native leaves (Figure 3). Transcription levels of *sdiA* on NO-excess and native environments were significantly different (*p* = 0.05). The multiple antibiotic resistance transcriptional regulator *marA* (Lee et al., 2015) was also significantly up-regulated in NO-excess and native plant environments compared to expression in the inoculum. This gene expression pattern suggests these genes may be important for leaf colonization. The outer membrane porin *nmpC* involved in H_2_O_2_ and other small molecule diffusion across cell membranes (Calderón et al., 2011) and *trpE*, a component of tryptophan biosynthesis, displayed uniform significant up-regulation in all treatments compared to inoculum (*p* < 0.05), indicating they may not be directly affected by plant derived NO stress.

### Expression of NO and ROS Detoxification Genes Was Detected in *Se*N Colonizing Fruit Surface

**FIGURE 4:**
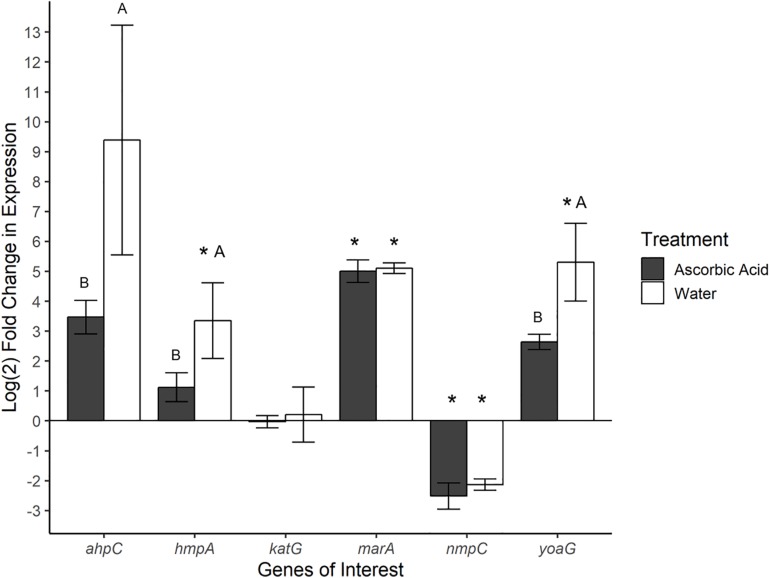
Log_2_-fold change in gene expression of *S.* Newport genes compared to expression in inoculum. ΔΔCt results of *S.* Newport colonizing tomato fruit pretreated to reflect the native fruit environment (H_2_O) or ROS-limiting (AscA) environments normalized against expression of sigma factor *rpoD.* Error bars represent the standard error of the mean. Asterisks denote significance in gene transcription compared to inoculum via Dunnett’s test (α = 0.05). Letters denote significant differences in expression between treatments for each target gene (Student’s *t*-test, α = 0.05).

Gene expression was also investigated in *Se*N associating with the surface of tomato fruit. Ascorbic acid was employed as an ROS scavenger (ROS-limiting environment) before challenging fruit with *Se*N and comparing expression profiles to *Se*N on water treated tomato fruit (native environment). In total, 72% of *Se*N on fruit samples displayed a ≥|2| -fold change in gene expression compared to the inoculum (Figure 4). *Se*N on native fruit exhibited significant up-regulation of *hmpA* and *yoaG* compared to the inoculum (*p* < 0.05). These genes were also up-regulated in the native fruit environment compared to the ROS-limiting environment (*p* < 0.05). Transcription of the oxidative stress gene *ahpC* was several log fold-change higher on native versus ascorbic-acid treated fruit (*p* < 0.05). The findings suggest *Se*N on fruit was countering plant derived NO stress and provides evidence that NO induction by *Se*N may be affected by ROS activity.

As seen in *Se*N on leaves, transcription levels of *marA* were higher in *Se*N on native and ROS-scavenged fruit (*p* < 0.05) compared to inoculum and appeared unaffected by modulation of ROS. Down-regulation of *nmpC* was detected in *Se*N on both fruit treatments (*p* < 0.05), compared to inoculum.

### Modulating Tomato Surface NO Levels Significantly Affected *Se*N Colonization of Leaves, but Not Fruit

**FIGURE 5:**
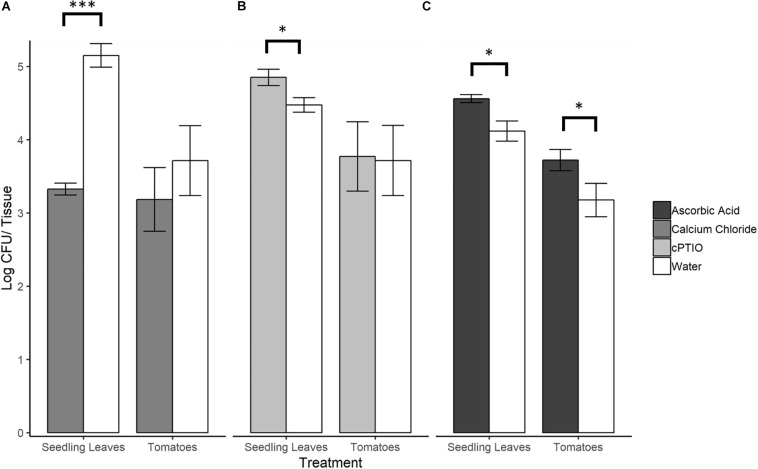
*S.* Newport counts retrieved from tomato seedling leaves and fruit after pre-treatment of plant tissue with **(A)** NO elicitor, **(B)** NO scavenger, or **(C)** H_2_O_2_ scavenger. Error bars represent standard error of the mean. Asterisks denote statistically significant difference by Student’s *t*-test between treatments and water control (α = 0.05) with ^∗∗∗^*p* < 0.001 and ^∗^*p* ≤ 0.05.

To evaluate whether *Se*N colonization of tomato surfaces was significantly affected by modulated levels of plant-derived H_2_O_2_ and NO, a series of 12 h on-plant *Se*N challenge assays were conducted on leaves and fruit. *Se*N counts recovered from leaves pre-elicited to produce endogenous NO were almost 2 log lower at 12 hpi (*p* < 0.001), measured at 3.32 ± 0.2 log CFU/leaflet, compared to 5.15 ± 0.3 log CFU/leaflet recovered from mock-treated leaves (Figure 5A). Conversely, *Se*N counts recovered from NO-scavenged leaves were higher (4.85 ± 0.5 log CFU/leaflet) compared to sterile water treated leaves (*p* < 0.05; Figure 5B). This effect was not observed on fruit. Regardless of pre-treatment, *Se*N was retrieved at higher titers with smaller coefficients of variation (CV), on leaf compared to fruit samples, in both NO scavenged tissue (CV_fruit_ = 0.39 and CV_leaves_ = 0.10) and NO elicited tissue (CV_fruit_ = 0.42 and CV_leaves_ = 0.24).

### Scavenging H_2_O_2_ on Tomato Leaves and Fruit Favors *Se*N

When ROS was scavenged from plant leaf and fruit samples with ascorbic acid, significantly higher *Se*N counts were retrieved from scavenged leaves (4.56 ± 0.10 log CFU/leaflet) and fruit surfaces (3.27 ± 0.47 log CFU/fruit) compared to control (*p* < 0.05; Figure 5C). On fruit, regardless of treatment, *Se*N retrieval was ∼0.8 log CFU/unit lower than on leaves, with a larger coefficient of variation (CV) in retrieval from fruit compared to leaves (CV_fruit_ = 0.20 and CV_leaves_ = 0.07).

## Discussion

In the present study, biochemical, gene expression and on-plant colonization assays of both vegetative tissue and ripe mature fruit provide evidence to support tomato plant recognition and response to *Salmonella* Newport. Tomato leaves and fruit generated ROS and leaves also generated NO in response to the enteric pathogen. In turn, *Salmonella* interpreted the mounted plant response as a stress, evidenced by differential ROS and NO detoxification gene up-regulation in *Salmonella* colonizing plant surfaces. *Salmonella*, therefore, may need to respond to plant-derived stimuli to ensure successful epiphytic colonization. Importantly, this study shows that tomato plants possess mechanisms capable of restricting *Salmonella* populations on leaf and fruit surfaces. ROS was detected in both leaf and fruit samples, and the reciprocal bacterial response was consistent with ROS negatively impacting *Salmonella* colonization, restricting bacterial counts. NO induction and an adverse effect of NO on *Salmonella* colonization was also detected on leaves, but not fruit. The ROS and NO bursts induced *Salmonella* to express genes needed for ROS and NO detoxification on leaves and NO detoxification on fruit. Despite no detected NO in fruit tissue subsequent to *Salmonella* challenge, and no effect of NO on bacterial restriction on fruit, bacterial gene expression results suggested *Salmonella* perception of NO on fruit. This work provides evidence that plant-derived NO is generated in response to *S. enterica* recognition.

Nitric oxide is required for PAMP-induced stomatal closure (Melotto et al., 2006). The production of NO in tomato leaves in response to *Salmonella* could therefore be signaling this innate immune response. This may also explain why NO was detected on leaves but not fruit, as tomato fruit lack stomata (Rančić et al., 2010). Low levels of NO, however, may have been present even on fruit, as transcription of NsR regulon genes was detected in *Salmonella* on both leaves and fruit. Up-regulation of NO detoxification gene *hmpA* during tomato surface colonization indicated that *S.* Newport perceived plant-derived NO as a stressor, corroborated by lower bacterial counts of *S.* Newport on NO-elicited seedling leaves. The NsrR regulon, controlled by the nitric oxide sensing transcriptional repressor NsrR, plays an important role in nitrosative stress resistance during infection. Within this regulon, the flavohemoglobin HmpA is identified as the main protein responsible for NO detoxification activities in the presence of an oxygenated environment (Karlinsey et al., 2012). A transcriptomic study of *S*. Typhimurium on tomato leaves and roots identified multiple up-regulated NsrR regulon genes, *ygbA*, *ytfE*, *yoaG* STM1808 and *yfhH* (Han et al., unpublished), which in *E. coli* is known to offer an NsrR binding site (Partridge et al., 2009). Supporting this, we assayed two genes in the NsrR regulon, *hmpA* and *yoaG*, both of which were differentially up-regulated compared to all native fruit and leaf environments. Han et al. (unpublished) measured gene expression in cells that had been colonizing plants for multiple days and did not detect *hmpA.* In our study, gene expression was assayed following 6 h of plant association, to capture *Se*N responses to early MTI. In fact, *hmpA* expression implies an early release of NO by the plant, and the need for *S*. Newport to mitigate its immediate effects. The NO detoxification-associated genes *hmpA, nfrA* and *ygbA* were also found to be up-regulated in soft rot macerated cilantro and lettuce leaf tissue caused by the plant pathogen *Dickeya dadantii* (Goudeau et al., 2013). In the present study, *Salmonella* NO detoxification was reported in the absence of a plant pathogen or tissue injury. Tomato plants produced NO upon perception of *Salmonella* which, in turn, led the bacteria to switch on reactive nitrogen species (RNS) detoxification machinery. This action may be necessary in order for *Salmonella* to successfully persist on some plant surfaces.

Evidence of ROS elicitation in tomato colonized with *S.* Newport was also strong, consistent with other reports of MTI induction in plants associating with this enteropathogen (Schikora et al., 2008; Shirron and Yaron, 2011; Meng et al., 2013; Garcia et al., 2014). The response we detected on leaves was diffuse across the leaf tissue and similar to staining reported for avirulent pathogens, as opposed to virulent pathogens (Großkinsky et al., 2012). Although *Salmonella* is not known to enter the apoplastic space (Potnis et al., 2014), the assay we used required infiltration, such that the response may have been more pronounced than what occurs when *Salmonella* is associating with the leaf epiphytically or residing in sub-stomatal chambers. In any case, ROS had a restrictive effect on *Salmonella* populations on the surface of both leaves and fruit and evidence of bacterial detoxification of ROS stress while colonizing leaf surfaces was detected via up-regulation of *S. enterica ahpC*. By contrast, we did not detect expression of the catalase *katG.* AhpC and the catalase encoded by *katG* are both known to scavenge H_2_O_2_ and organic hydroperoxides, but we only detected consistent expression of *ahpC* in *Salmonella* associated with leaves. In macrophages, *ahpC* expression is stronger than *katG* expression (Hébrard et al., 2009). Ahp was reported to be a more efficient scavenger at low concentrations of H_2_O_2_ (below 20 μM) than catalase, which becomes the primary active enzyme at higher H_2_O_2_ concentrations (Seaver and Imlay, 2001). From our findings, therefore, we infer that levels of H_2_O_2_ reaching *Salmonella* on the tomato plant surface were in concentrations insufficient to induce catalase activity.

Modulation of NO on leaves did not impact *ahpC* expression, but the use of an ROS scavenger on fruit reduced transcription of *ahpC.* Transcription of the NsrR-regulated *hmpA* and *yoaG* genes in *Salmonella* on fruit was also higher when ROS was not attenuated, suggesting that higher levels of ROS may be related to NO levels. A large degree of interconnectivity exists between NO and ROS signaling in plant tissue (Romero-Puertas and Sandalio, 2016). On native and ascorbic acid-treated fruit tissue, *Salmonella* would have been responding to NO levels induced by the enteropathogen itself. However, NO has been shown to regulate ascorbate peroxidase (APX) (Yang et al., 2015), the enzyme that uses ascorbate as an electron donor to reduce H_2_O_2_ to H_2_O. On ascorbic acid-treated fruit, therefore, the depleted H_2_O_2_ environment could have signaled the attenuation of NO, such that *Salmonella* would have been responding to lower NO levels reflected in reduced *hmpA* and *yoaG* transcription.

Other than NO and ROS stress, *Se*N gene expression on leaves and fruit was indicative of adaptation to a novel environment. In leaves, *trpE*, a gene in the tryptophan biosynthesis pathway which has been associated with biofilm development (Hamilton et al., 2009), was up-regulated in all environments. Biofilm formation is known to enhance the capacity of pathogenic bacteria to survive stresses in the environment and during host infection. Thus, the present work provides more evidence to the growing body of work which defines attachment as paramount to survival in the phyllosphere (Barak et al., 2005, 2007). Also up-regulated in all leaf and fruit environments was the gene *marA*. In addition to their importance as regulators of xenobiotic efflux, *marRAB* may have indirect effects on expression of iron metabolism genes, membrane composition genes, and the stress related sigma factor *rpoS* (Lee et al., 2015). In *S. enterica* transcriptomic surveys (Wang et al., 2010; Brankatschk et al., 2014), the use of such machinery was found to be pertinent to survival in stress-inducing environments. In our study, *nmpC* (*ompD*, STM1572) displayed differential expression among plant tissue types, up-regulated compared to inoculum in *Salmonella* on leaf surfaces but down-regulated on fruit surfaces. NmpC is one of the most abundant outer membrane porins of *S. enterica*, used to transport molecules, including H_2_O_2_, into the cell and toxins out of the cell (Calderón et al., 2011). Expression of *nmpC* was down-regulated in *S.* Typhimurium exposed to H_2_O_2_ (Calderón et al., 2011). Additionally, NmpC may be needed for adherence and recognition of *S*. Typhimurium to human macrophages and epithelial cells during the initial stages of infection (Hara-Kaonga and Pistole, 2004) and is also involved in host cell recognition in mammalian models (Ipinza et al., 2014; van der Heijden et al., 2016). In our study, we observed up-regulation of both *nmpC* and ROS detoxification gene *ahpC* on leaves. However, *nmpC* was down-regulated in *Se*N on native fruit where *ahpC* expression was more variable. These findings suggest an additional function of this porin, perhaps engaging in efflux activity of other xenobiotics. Taken together, targeted gene expression on both leaves and fruit provide evidence that *S. enterica* attaches to and recognizes various stressors on plant surfaces.

Working with NO modulation on fruit proved challenging. While up-regulation of bacterial NO detoxification genes was detected on fruit, we could not easily measure NO released from fruit tissue challenge with various biotic treatments. *Salmonella* produced a fluorescent response in fruit exocarp greater than that of leaves but was not consistent. It is possible that exocarp excision generated high levels of NO, even in the negative control, and masking the weaker signal elicited by the pathogen. This could also explain why DAF-2 DA fluorescence diminished over time, even in the positive control. Further, ascorbic acid significantly affected *S. enterica* counts on fruit whereas NO modulation did not. This could be attributed to NO and ROS endogenous levels in mature red fruit at the time of study. Ripe red fruit are known to have lower concentrations of nitric oxide compared to mature green fruit, as NO is involved in regulating ethylene production and thus facilitating the ripening process (Ya’acov et al., 1998). Most NO modulation studies in fruit responding to plant pathogens are routinely conducted with mature green fruit (Lai et al., 2011; Zheng et al., 2011a, b; Zhu and Tian, 2012). The transition from green to red fruit is marked by accruement of high ROS concentrations (Kumar et al., 2016). Petrasch et al. (2019) investigated ROS detoxification by fungal pathogens in red ripe fruit. Thus, the ascorbic acid injected in our plant colonization assays could have been targeting ripening-related ROS, hence providing a more hospitable environment for colonizing *S. enterica*. Mature ripe fruit tissue also has lower levels of pathogen recognition response capacity compared to vegetative tissue, perhaps due in part to the breakdown of cellular wall components during ripening (Cantu et al., 2009). *S. enterica* studies on ripe and unripe tomato fruit have found the organism proliferates more readily in ripe red compared to mature green tomato fruit (Barak et al., 2011). Taken together, these data imply mechanisms of *S. enterica* restriction are plant tissue-specific and may be facilitated or confounded by the fruit ripening process. Regardless, more research is needed to evaluate the interconnectivity between ripening and pathogen defense, both in the contexts of plant and human pathogens.

Overall, higher titers of *S*. Newport were consistently retrieved from leaves compared to fruit, an observation which has been reported elsewhere (Barak et al., 2011; Han and Micallef, 2014; Gu et al., 2018). Supporting evidence can be found in *Salmonella* field sampling studies. For example in a multi-year field study, sampling tomato leaves and fruit for wild *S. enterica* colonization found only leaves returned positive *S. enterica* result, never fruit (Gu et al., 2018). This tissue-specific variability in *Salmonella* carrying capacity could be due to relative abundance and composition of nutrients on the surface of the different plant organs, higher relative humidity on leaves as a result of transpiration, and a higher and more rugged surface area or attachment on leaves compared to fruit. Higher proportions of fatty acids have been seen in fruit washes of tomato cv. ‘Heinz-1706’ compared to seedling shoot or mature leaf washes, and this correlated negatively with *S. enterica* growth (Han and Micallef, 2016). While the leaves of the tomato are not eaten, in the field leaves and tomato fruit are in constant contact with one another, serving as a contamination source for fruit. Vegetative matter is commonly picked up during harvest of tomato fruit and could lead to widespread contamination if appropriate Good Agricultural Practices are not followed. Cross-contamination of fruit from contaminated vegetative matter is a potential risk when using recirculating water to wash tomatoes without the appropriate concentration of sanitizer (Bolten et al., 2020).

We noted that *Se*N retrieval from leaves was less variable than fruit, suggesting a potential heterogeneity of response to unique stressors present on the latter plant organ. Diversification of stress response or “bet hedging” has been documented in intercellular *S. enterica* interaction with ROS and other stress agents (Helaine and Holden, 2013; Burton et al., 2014; Helaine et al., 2014). Tomato fruit may be a uniquely harsh plant niche for *S. enterica* for which “bet hedging” may be a significant strategy to ensure long term survival. Understanding tissue specific enteropathogen–plant interactions therefore can help devise strategies to minimize fruit contamination. Yet, enteropathogen–plant interaction data relevant to agricultural situations, which relate directly to salmonellosis outbreak-causing *S. enterica* strains, remain limited.

*Salmonella enterica* mitigation of host-derived NO and ROS is crucial for successful invasion in animal host models. These processes have been well documented (Vazquez-Torres and Fang, 2001; Zheng et al., 2011a; Spector and Kenyon, 2012; van der Heijden et al., 2015). Presence of RNS in the mammalian gut increased overall colonization fitness of *S. enterica*, possibly because it can outcompete some resident microflora (Stecher et al., 2007). This compounds the importance of investigating the presence of analogous interactions in *S. enterica–*plant associations. Perception and response to NO and ROS, which can be short-term restricting agents, together with other important stress and host adaptation responses, may lead to long-term persistence in the field. As ROS and RNS may be present in multiple scenarios in the agricultural setting (Diaz and Plummer, 2018), mitigating these compounds may be a key factor for *S. enterica* persistence in between entry into an animal host. Future work should investigate the ability of *Salmonella* to mitigate these stresses on plants and whether they are shared by all serovars, or specific to serovars that are regularly implicated in produce outbreaks. This is essential to continue to elucidate *Salmonella* adaptation to non-animal host environments.

Deciphering the highly nuanced and complex plant-associated lifestyle of this enteric pathogen is imperative to inform strategies to minimize successful *Salmonella* contamination and persistence in an agricultural setting and help in identifying plant traits or cultivars that are unfavorable for *Salmonella* colonization. Furthermore, how non-plant pathogenic microorganisms, including enteric pathogens, interact with the plant immune system to colonize the phyllosphere is poorly understood. The MAMPs recognized by plants that result in MTI are highly conserved among microbes. It has recently been suggested that plants cannot differentiate between pathogens and commensals, and micro-organisms must evade or attenuate plant immunity to colonize plants (Teixeira et al., 2019). Untangling the role that plant immunity plays in the establishment of human pathogens on plants would add to knowledge about microbiome assembly, while also elucidating mechanisms that can lead to enhanced food safety.

## Data Availability Statement

The datasets generated for this study are available on request to the corresponding author.

## Author Contributions

SM and AF conceptualized the study and designed the experiments. AF conducted the experiments. AF, SB, and BS designed and conducted q-RT-PCR experiments. AF and SM analyzed and interpreted the data and wrote the manuscript. SB and BS reviewed and edited the manuscript. SM administered the project.

## Conflict of Interest

The authors declare that the research was conducted in the absence of any commercial or financial relationships that could be construed as a potential conflict of interest.
