# Supplementary material for: Salmonella enterica Elicits and Is Restricted by Nitric Oxide and Reactive Oxygen Species on Tomato
Source: Front Microbiol. 2020 Mar 13;11:391. doi: 10.3389/fmicb.2020.00391 (PMC7082413; doi:10.3389/fmicb.2020.00391)

Supplementary Figure 1

(A) Plate counts of 6 hour *S.* Newport incubation in Phosphate Buffered Saline amended with plant scavenger or elicitor of NO. (B) Plate counts of 6 h *S.* Newport incubation in TSB amended with plant scavenger or elicitor of NO. Inoculum was 7 Log CFU/mL for both A and B, no significant differences in growth after 6 h. (C) Gene expression of *S.* Newport *hmpA, katG and yoaG* genes relative to *rpoD*. *S.* Newport was grown in 1/2 strength TSB amended with $CaCl_2$ or cPTIO. All Log fold change values in expression are between 1 and -1.

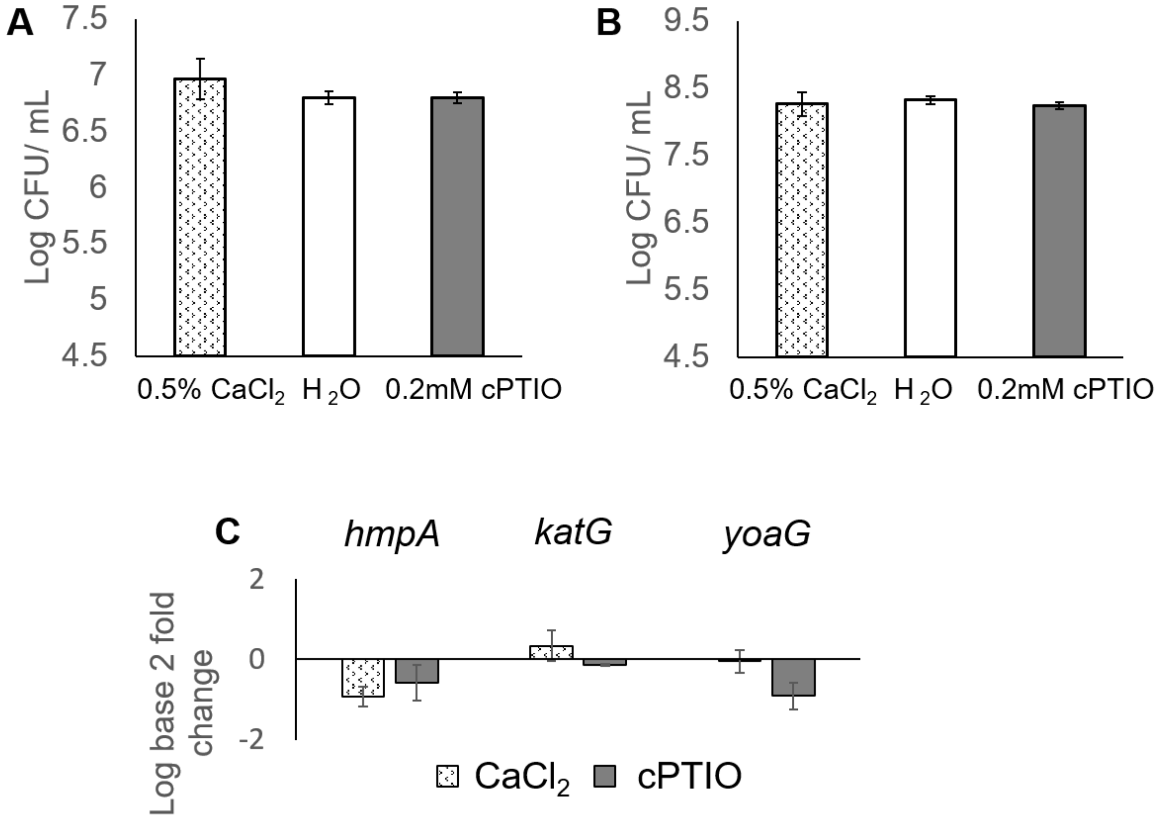

Supplement: Supplementary file 1 [file Data_Sheet_1.PDF]
